# Perceived Environmental, Individual and Social Factors of Long-Distance Collective Walking in Cities

**DOI:** 10.3390/ijerph15112458

**Published:** 2018-11-04

**Authors:** Peng Yang, Shanshan Dai, Honggang Xu, Peng Ju

**Affiliations:** 1 School of Tourism Management, Sun Yat-sen University, Zhuhai 519000, China; yangp55@mail2.sysu.edu.cn (P.Y.); xuhongg@mail.sysu.edu.cn (H.X.); 2 Shenzhen Tourism College, Jinan University, Shenzhen 518053, China; jupeng@sz.jnu.edu.cn

**Keywords:** well-being experience, long-distance walking, collective leisure activity, walking event, urban leisure

## Abstract

Long-distance collective walking is a popular activity in cities across China. However, related research is limited, creating a research gap to explore participants’ dynamic experience and related influential factors. Therapeutic mobilities theory explores the relationships among walking, health, and well-being from a qualitative perspective. Based on therapeutic mobilities theory, following a systematic process, this study develops a scale to quantitatively estimate the perceived environmental, personal, and social factors that may influence health and well-being. By applying construal level theory, this paper further hypothesizes that personality traits and familiarity moderate environmental, personal, and social perceptions. Data were collected with a paper survey (*n* = 926) from the “Shenzhen 100 km Walking” event. The findings highlight that long-distance collective walkers have comparatively greater experiences of health and well-being in three aspects: positive social interaction, individual development, and environmental understanding. Personality traits, familiarity, and gender moderate this well-being experience. Theoretical and managerial implications are discussed.

## 1. Introduction

Walking is diverse and dynamic [1]. In the past two centuries, walking has shifted from a central mode of transport to a leisure activity [2]. In recent years in China, walking has become a popular daily leisure activity for urban residents. The number of people aged 20 and older who regularly participate in “fitness walking” reached 54.6% in 2014, an increase of 12.8% compared to 2007 [3].

Previous research has investigated diverse styles of walking, including wandering, strolling, trail-walking, trekking, hiking, and hiking-walking. Organized long-distance collective walking (LDCW) is a newly developed walking event that has spread widely in Chinese cities. Previous researchers have focused on the emotional experience and health function of walking [2,4,5,6,7]. However, as long-distance collective walking is a walking event, physical health factors may not be the main concern. The impact of walking on well-being, as an emerging research direction, provides a new perspective to understand why people engage in long-distance walking [8,9].

Gatrell (2013) proposed the therapeutic mobilities theory to map the relationships between walking and well-being and health from a qualitative approach. Based on the therapeutic mobilities theory, this study develops and validates a scale to estimate the subjective health and well-being experiences of long-distance walkers. To our knowledge, this study is the first attempt to examine empirically the participants’ experience within a walking event context.

By applying construal level theory [10,11,12,13], this paper investigates the factors that influence the experience. The moderating roles of adventurous personality traits, familiarity, and gender are explored. Data from the “Shenzhen 100 km Walking” event, which is a well-known LDCW activity in China, were collected and used in this empirical study. This study will provide managerial insights for walking event managers and urban planning officials to design walkable environments and target different population segments.

## 2. Literature Review

### 2.1. Walking, Experience, and Well-Being

Exploring comprehensive experiences provides a foundation for understanding complex tourism and walking activity [14,15,16,17]. Donald and Vesna (2009), through hiking and walking in Mountain Nature Park, identified three main experiences: (1) affinity with nature and the outdoors, (2) mental and physical benefits, and (3) interaction with others and development of self-knowledge [18]. Their findings are supported by various studies [19].

Specifically, Gatrell’s (2013) framework for walking components serves as a foundation for structuring the relationship between walking experiences and well-being [20]. Based on his idea that movement itself can be conducive to well-being and health, and a literature review, he argued that walking contributes to well-being through three aspects: It improves physical fitness and mental health, it cements existing or develops new friendships and social interactions, and it permits an engagement with places and environments as encountered on the move. He named these three aspects the active body, the social body, and the walking context, respectively.

Engaging in physical activity, walkers may have individual physical and psychological experiences. Walking is generally acknowledged as the most common form of exercise. Regular walking of moderate to vigorous intensity is the traditional research focus in the walking field [21,22]. Regular walking is among the most effective interventions when used to promote physical activity and adherence to exercise [21]. Morgan, Tobar, and Snyder’s (2010) comparative study of walkers indicated that walking can benefit both cardiovascular and psychological health [22]. Psychological benefits include improved sense of well-being, more positive (i.e., vigor) and less negative (i.e., tension, depression) feelings and mood states, and enhanced self-esteem [23,24].

Walking is inherently a social activity [25]; different types of social relations are identified as arising from the walk experience [26]. For example, many walkers share a social experience which is similar to a festival experience [27]. Walking helps to develop social connections with other people. Walking is a way “to go out to be energized by different people” [28]. In the city, walking improves the levels of social interaction and participation in neighborhood life. If the environment is perceived as safe and friendly, people are more likely to engage with others, including volunteering and attending activities in local community centers [29].

Walking also provides an opportunity to be aware of one’s surroundings [20]. The environment has an important impact on walking [30]. Walking activities usually happen in specific environment settings, such as, for example, natural areas [31,32], the countryside [2]), the urban environment [5,33,34], and trails in parks [18], as well as other areas [31]. Some studies have highlighted the experience with nature, such as a wilderness experience. The wilderness experience embodies such aspects as autonomy, spontaneity, solitude, freedom of action, challenge, risk, spiritual values, and aesthetic appreciation [35,36]. Other studies have explored the effective potential of walking in the full range of typically encountered non-natural built settings, specifically, urban settings. Bornioli, Parkhurst, and Morgan (2018) showed that walking in high-quality urban settings can have positive outcomes. However, the walking environment of LDCW is complicated and includes not only urban and rural areas, but also natural areas.

Consumers’ feelings affect their quality of life [37] and the environment critically influences the health and well-being of a city’s inhabitants [38]. Recent studies have explicitly linked walking and well-being [9,20,28,39,40]. Morgan, Tobar, and Snyder’s (2010) research indicated that continuous walking positively influenced a number of variables that are indicators of physical and psychological well-being [22]. Doughty’s (2013) ethnographic study investigated the social dynamics of embodied movement in a walking group and found therapeutic outcomes [39]. Furthermore, because the act of walking includes interaction with the physical landscape and social surroundings (whether intended or unintended), studies have encouraged the mobilization of the “therapeutic landscapes” concept to better grasp the interconnections of walking, well-being, and place [20,39]. Walking is a kind of therapeutic mobility step to well-being.

### 2.2. Long-Distance Collective Walking

Few studies have explored the experience of long-distance walkers [14,15]. Long-distance walking refers to either single-day walks of 20 mi (about 32.2 km) or more or multi-day walks that typically follow designated long-distance footpaths [1].These activities have the following features: recreational and long-distance walking in multiple environments including urban and natural environments, organized by volunteers or non-governmental organizations (NGOs), and generally in small groups but with overall numbers reaching more than 10,000. The meaning of long-distance walking goes far beyond the physical and psychological. The long-distance route transforms recreational walking into a multi-day holiday.

Related research has highlighted the happiness experience, such as enjoyment and engagement [14,15,16]. Seven experience items have been identified, including enjoy meeting fellow walkers, experience of solitude, experience of freedom, having time to think and relax, enjoy the scenery, and feeling closeness with nature [14]. Rather than decreasing in intensity, the enjoyment of long-distance walking finishes on an upward trend. Saunders, Laing, and Weiler (2013) interviewed 25 long-distance walkers reporting personally significant experiences on multi-day hikes, suggesting increased self-confidence and other enduring changes which enhance well-being [41]. Crust, Keegan, Piggott, and Swann (2011) aimed to understand walkers’ positive psychological movement from three aspects: life of enjoyment, life of engagement, and life of affiliation. This study was conducted in a natural space away from the urban environment [15]. Based on his investigation with six long-distance walkers, the essence of long-distance walking is described as a “journey of self-discovery” that occurs within a world detached from the stresses of modern life. Compared to regular walking or a sport event, long-distance walking might come with higher intensity and greater mental challenge and result in a flow experience and engagement.

While researchers have found that social interaction is a vital and enjoyable aspect of shared experience [15], the social interaction of long-distance collective walkers has not been fully discussed. In the Western context, many walkers walk alone. Walking is often regarded as an individual activity and demonstrates its effectiveness as a physical and psychological treatment activity [21,22,32,42,43]. Some studies have examined solitary walkers [32,42,43,44] or small group walking practices [45]. Some walkers prefer to enjoy an individual solitary experience. For them, the walking environment just provides a bubble for a “journey of self-discovery” [15]. Wylie (2005) added that walking alone allowed “a close visual, tactile, and sonorous relationship with the earth, the ground, mud, stinging vegetation” [43]. Since collective walking is a particular walking style, walking group studies have thus far shown evidence that group walks provide an excellent milieu in which social networks can be generated and strengthened [46]. Outdoor group walks also have the potential to be a useful health intervention as they increase physical activity and are cost effective [47]. Walking provides opportunities for stimulation, restoration, contemplation [40], and in the case of collective walks, a sense of pleasure from the shared experience [48]. Furthermore, in the Eastern context, people may prefer walking in a group because the collective preference may be more important; this will be tested in our study.

Research has documented the different kinds of walking experiences in different contexts. A substantial body of research on walking exists and there are many types of walking and many areas and environmental conditions in which walking is, or can be, performed. Among these studies, relatively few have focused specifically on walking as a collective activity. At the present time, no single theory seems capable of explaining the experience of LDCW and the links between the walkers’ experience and their well-being. Thus, the argument goes that it is not so much the inherent and perceived properties of walking that matter, but rather the experiences walkers get from LDCW in the Eastern context and how the experiences contribute to the walkers’ well-being.

Gartrell’s therapeutic mobilities theory has been extensively employed in exploring casual walkers’ experience. Therapeutic mobilities theory maps the relations between walking and well-being and health using a qualitative approach. The core of the theory is that walking is therapeutic in the active body, social body, and walking context [20]. These three aspects shape the characteristics of walking. The number of participants in long-distance collective walking events is relatively high. Empirically estimating LDCW participants’ experience may provide an opportunity to understand why LDCW is popular. On the basis of Gartrell’s theoretical framework, walking is therapeutic in that the active body provides a physical and emotional aspect experience, walking is therapeutic in that the social body provides a social experience, and the walking context provides an environmental experience. Thus, we propose the following hypothesis.

**Hypothesis** **1** **(H1)** *The experience of long-distance collective walkers includes three aspect of well-being experiences: physical and emotional experience, social experience, and environmental experience.*

### 2.3. The Moderating Effect of Personal Traits and Familiarity Based on Construal Level Theory

Construal level theory is a social psychology theory that describes how the context, such as the psychological distance, shapes mental representations [10,11,12]. Researchers have shown that different dimensions of psychological distance affect mental construals [49]. According to the theory, peoples’ temporal perspectives influence how they evaluate an event [11] and therefore might affect their experience. An individual will likely view a far-distant event in abstract terms, consider general issues, and describe the event using dream-like words. In contrast, a near-distant event is viewed in more concrete terms and in greater detail, with more practical issues being considered [13].

Construal level theory is powerful in explaining consumer behavior and perception. However, it has received limited attention in walking and well-being research. Walking participants are heterogeneous, and different walkers have different experiences [1]. Within the context of LDCW, participants’ sensation-seeking personality and familiarity with LDCW represent their psychological distance [50].

Personality traits determine the tendency to seek various experiences and sensations and the willingness to obtain stimulation [51]. Personality may influence destination choices, leisure activities, and other travel-related decisions [52]. Based on construal level theory, people’s psychological experience of something is egocentric, specifically influenced by the level of mental construal. This egocentric mental construal is characterized by personality traits in this study. In the specific case of long-distance walking, which is a kind of adventure activity, participants with adventure-seeking tendencies may seek novel, varying, and stimulating experiences. Adventure-seeking is an often recognized and studied sub-dimension of personality traits [53]. Accordingly, we propose the following hypothesis.

**Hypothesis** **2** **(H2)** *In the context of LDCW, walkers with higher adventure-seeking tendencies have more well-being experiences*.

**Hypothesis** **3** **(H3)** *In the context of LDCW, walkers with higher adventure-seeking tendencies have more environmental experiences*.

**Hypothesis** **4** **(H4)** *In the context of LDCW, walkers with higher adventure-seeking tendencies have stronger individual experiences.*

**Hypothesis** **5** **(H5)** *In the context of LDCW, walkers with higher adventure-seeking tendencies have stronger social experiences*.

Well-being effects derived from a walking environment may depend on personal characteristics such as age, gender, and physical condition [40]. Social factors or socio-demographic attributes are significant covariates of urban residents’ mental health [54]; thus, gender is another factor that may moderate the LDCW well-being experience. Because of their longstanding social roles and social identities, men and women have different physical activity behaviors [55,56]. Overall, women spend considerably more time walking than men [57] and more women than men walk for errands and leisure, in line with a general trend for women to devote more time and make more trips than men to serve their household [58]. The level of physical activity also differs by gender, with women being less active than men [59]. In addition, men often outperform women in physical activities, but women’s emotional and psychological experiences in leisure activities are more intense. Women tend to be more sensitive to their environment [60]. Within the LDCW context, we propose the following hypothesis.

**Hypothesis** **6** **(H6)** *In the context of LDCW, walkers’ gender affects the experiences of well-being*.

**Hypothesis** **7** **(H7)** *In the context of LDCW, female walkers have stronger environmental experiences*.

**Hypothesis** **8** **(H8)** *In the context of LDCW, female walkers havefewer individual experiences*.

**Hypothesis** **9** **(H9)** *In the context of LDCW, female walkers have fewer social experiences.*

Construal level theory also points out that psychological experience is determined by time, space, and social and hypothetical distance [13]. When people have high familiarity with a particular activity, the time distance between them is shorter, the space distance is closer, and the social distance is closer. As to LDCW, some researchers have pointed out that walking in unfamiliar environments may result in negative emotional experiences, such as feelings of solitude [61], fear [32], depression, tension, isolation, or being confined [31], and the familiarity that walkers have with the environment and activity has an impact on their experience. Familiarity in a commercial sense usually refers to the cumulative number of times a consumer experiences a product and is related to the number of times consumers use the product [62,63]. Accordingly, we propose the following hypothesis:

**Hypothesis** **10** **(H10)** *Walkers’ familiarity with LDCW moderates their well-being experiences.*

**Hypothesis** **11** **(H11)** *Walkers who have higher familiarity with LDCW have lower environmental experiences.*

**Hypothesis** **12** **(H12)** *Walkers who have higher familiarity with LDCW have higher individual experiences*.

**Hypothesis** **13** **(H13)** *Walkers who have higher familiarity with LDCW havefewer social experiences*.

## 3. Method

### 3.1. Shenzhen 100 km Walking

**Figure 1:**
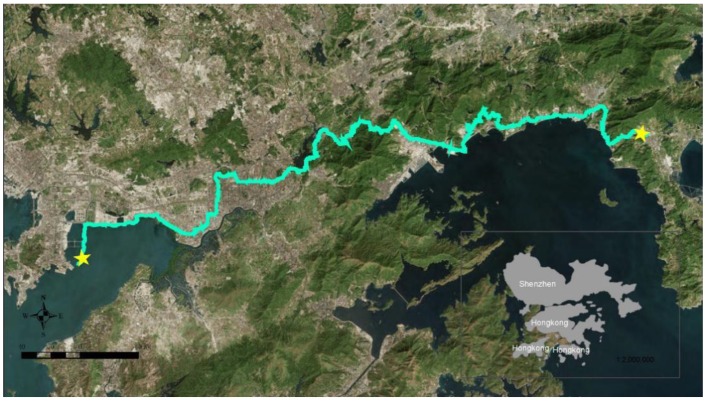
The walking trajectory of the 2016 “Shenzhen 100 km Walking” event.

The “Shenzhen 100 km Walking” event is held by MoFang Forum, a famous outdoor network platform in China. The “Shenzhen 100 km Walking” event is one of the most representative of many large-scale walking events in China and was one of the first walking events in China. The first event was held in 2001, and Jin (2012) pointed out that from the beginning of 1998, walking events only emerged in Beijing, Guangzhou, Kunming, Shanghai, and other large cities, and various walking events organized by governments only began to emerge on a large scale after 2005 [64]. The “Shenzhen 100 km Walking” event has been held 16 times, and it has a broad social impact. The number of participants in the first session was 52, and in 2016, the number of participators formally signed up was 60,723; the actual number of participators was more than 100,000. The walking trajectory of the 2016 event is shown in Figure 1. The walking trajectory is along the southern border of Shenzhen.

**Figure 2:**
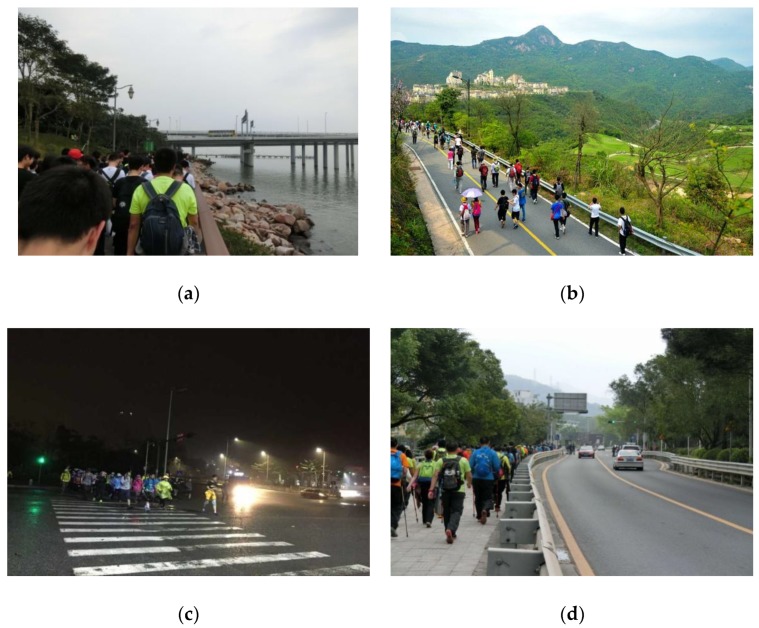
The typical walking environment of the 2016 “Shenzhen 100 km Walking” event [65]: (**a**): Ocean Bay; (**b**): mountain area; (**c**): urban area (walking in the night); (**d**): rural area.

First, the “Shenzhen 100 km Walking” is a non-competitive long-distance walking event in which participants walk about 100 km within one day, with the hiking routes set up by organizers within the multiple environments of Shenzhen city. The typical walking environments are shown in Figure 2. Not everyone is required to complete the whole 100 km; the organizers believe that participants should choose the distance to walk based on their own abilities. Most of the participants are hiking enthusiasts and some just want to feel the atmosphere of the event. Organizers have stressed that participants enjoy the event as it eases the pressures of life and promotes a healthy and environmentally friendly way of life.

Second, it is a collective activity. It is a large event initiated by tour pals (travel enthusiasts) [66] in the network platform MoFang. MoFang users themselves set up the organizing committee, organize activities, and organize this event. Formal participants need to join a team of 5–6 people to complete the registration, and many informal participants join the walking teams during the event. The participants are not only citizens of Shenzhen, but people from all parts of China. Some media have called it a “folk organization’s walking event [67].”

Third, it is a meaningful human mobility event. Each session of the “100 km” event has a clear theme. For example, the first session is themed “Feet Measured Shenzhen,” the thirteenth session is themed “Walk without Leaving a Trace,” and the sixteenth session is themed “Let’s Go,” which reflects the cultural theme of these outdoor activities and the main form of this event, which is a kind of human mobility. This event has become a “very influential business card of Shenzhen” [68] and has even “become a side of the cultural banner of Shenzhen” [69].

### 3.2. Questionnaire Development

As the development and validation of a questionnaire require a systematic process [70] and no established measurements existed for a walking event, measures for the experience and relevance of an LDCW were developed specifically for this study.

Rigorous procedures for scale development were followed [71,72]. The first step was specifying the construct domain of the “LDCW experience.” In this stage, a focus group consisting of three experts and two PhD students majoring in leisure and event research was established to discuss and define the characteristics of a walking experience. Discussions combined with an extensive literature review helped us identify the physical aspect, emotional aspect, environmental aspect, and social aspect as four dimensions of a walking experience. After the construct domains were specified, both deductive and inductive approaches were used to generate an item pool to measure each dimension [73]. Four dimensions relating to the walking experience were collected and adapted from the literature to generate the initial items. This process resulted in the identification of 16 items: 3 in physical activity aspects, 6 in emotional aspects, 5 in environmental aspects, and 2 in social aspects.

Thereafter, semi-structured interviews with people who had participated in related activities were carried out. Based on the participants’ descriptions of their LDCW experiences, corresponding items were added or dropped in the questionnaire. Specifically, two items were added and two were dropped in emotional aspects, four items were added and two dropped in the environmental aspect, and two of the environmental aspects were divided into four items. Interviewees were also invited to assess the content validity of these items, asked to provide comments on the content and understandability of the items, and asked to edit and improve the items to enhance their clarity and readability.

To test the hypothesis that personality traits and familiarity affect the experience, the questionnaire also included adventure-seeking scales to measure participants’ self-cognitive assessments of personality characteristics. Adventure-seeking scales had two items (I like to do frightening things, and I would like to try bungee jumping) [74]. These two items were revised to I like outdoor activities”, according to a revised Chinese version developed by Chen et al. [53]. The second item was having adventures always makes me happy. Within the LDCW context, we used two questions to evaluate personality traits: I am a person who likes to participate in outdoor activities and I am a person who likes to challenge myself.

The participants’ familiarity with walking events was assessed by this question: “Have you enrolled in the event in advance?” As the route of a long-distance walking event changes every year, familiarity in the context of LDCW is defined by whether the participant has engaged in pre-trip planning [75]. In LDCW, not all participants are required to enroll in advance, but if the participants do enroll in advance, they need to be involved more in pre-walk planning, such as becoming familiar with the route, the check-in point, and the event theme.

The specific items are shown in Table 1. The initial set of 19 items was developed, and 5-point Likert scales ranging from 1 (strongly disagree) to 3 (neither agree nor disagree) to 5 (strongly agree) were used.

### 3.3. Pilot Study

After the content adequacy and validity were ensured, an initial questionnaire was designed and a small-scale pretest was conducted. The purpose of the pilot study was to determine whether our planned measures for the variables were meaningful to the respondents. The test was conducted on a group of participants (*n* = 105) who had attended the “2015 Shenzhen 100 km Walking” event and who were recruited through an online survey by snowball sampling. The participants were asked to rate the validated set of items.

This step was to explore the structure of the meaning of the experience and to test the concurrent validity. An exploratory factor analysis (EFA) was conducted using the data from the pretest, and the results are presented in Table 2. Before performing the EFA, the appropriateness of the 105 responses was examined. The normality was judged by estimating the skewness and kurtosis of each item. The slightly non-normal distribution is not likely to influence the final results [77].

Principal component analysis with varimax rotation was used for the exploratory analysis, and the structure was determined by the rotated component matrix. The number of factors was identified using the eigenvalue greater than 1.0 criterion [78]. The eigenvalue indicated that three common factors reflect the data characteristics well. Physical experience and emotional experience merged into one factor, and further reduction of items was performed. Items with high cross-loadings (>0.5) and low factor loadings (<0.5) were deleted one at a time to ensure accuracy. After each deletion, the Kaiser-Meyer-Olkin (KMO) value and commonalities were re-examined. After no item failed to meet the criteria, there were 16 measurement variables which were entered in the factor analysis (Table 2). In this step, three items were excluded: “bring health and fitness,” “stress release,” and “embrace trade-offs and compromise.” The internal validity was assessed. The KMO value was 0.858, which was close to 1, indicating sampling adequacy, and Bartlett’s test of sphericity was 965.578 (*df* = 120, *p* = 0.00 < 0.05), supporting the factorability of the data. Three factors explained 66.188% of the total variance (>cutoff value 60%). Cronbach’s alpha values ranged from 0.843 to 0.878 (>0.80), and the reliability of the questionnaire scale was established.

The pilot study showed three factors of the LDCW experience. Factor 1 involved items measuring environmental experiences (three items). The experience dimension is the most important factor (variance contribution rate is 25.196%). Factor 2 focused on the five items measuring individual experience, emphasis on physical cognition, and perceptions of positive mental experiences. The variance contribution rate of Factor 2 was 20.672%. Factor 3 contained the four items measuring the social interaction experience (Table 2).

### 3.4. Investigation Procedure

After discussing and resolving any discrepancies, the survey was considered to be appropriate for data collection. Formal research was conducted on 20 March 2016, during the “Shenzhen 100 km Walking” event, and 22 research assistants were sent to collect the questionnaires from the participants. As some participants might choose to withdraw halfway through, we chose two points, at the middle (Dongbei) and the end (Dapeng square) of the walking route to collect the data. Hard-copy questionnaires were used. Questionnaires were distributed face-to-face. A total of 1000 questionnaires was distributed with 926 valid questionnaires recovered, for a recovery rate of 92.6%; 328 valid questionnaires were collected in Dongbei and 598 valid questionnaires in Dapeng square.

## 4. Results

### 4.1. Respondents’ Profiles

To gain a preliminary understanding of the respondents, descriptive statistics were gathered, and the results are presented in Table 3. The profiles of the respondents showed that most walking event participants in this study were well educated (e.g., bachelor’s degree and above, 54.0%), young (under 35, 76.0%), and male (72.3%). This group of people also reflected the typical characteristics of the new generation in China which has a good education and the wealth to seek new experiences to complement and fit into their busy daily routines [23].

### 4.2. Model Evaluation and Scale Reliability

Confirmatory factor analysis (CFA) was used to verify factorial structures and items identified from the EFA. Before conducting the CFA, the data were screened to see if they violated multivariate normality by Holling’s *T* test [77]. Confirmatory factor analysis was conducted with the software AMOS 21.0, and the measurement properties of the 16-item scale were assessed by examining the overall model fit.

First, the overall model fit was evaluated. The goodness-of-fit indices include the overall fit index *χ*2(101) = 224.083, absolute goodness-of-fit index (GFI) = 0.923, value-added goodness-of-fit index (AGFI) = 0.889, normed fit index (NFI) = 0.910, comparative fit index (CFI) = 0.971, and incremental fit index (IFI) = 0.923, all of which were higher than the cutoff value 0.9; the root mean square error of approximation (RMSEA) = 0.075 was less than the ideal value of 0.1 [79]. The overall model fit indicated that the model fit the data adequately.

Subsequently, we assessed the reliability and validity of the identified scale. The standardized factor loadings of all factors were higher than 0.6, with the Cronbach’s α coefficients and combination reliability (CR) value of each factor being greater than 0.8 and average variance extracted (AVE) values higher than or close to 0.5 [79], indicating acceptable internal consistency. The final results are presented in Table 4. Discriminant validity is confirmed when the square root of the AVE exceeds the inter-correlations of the construct with other constructs in the measurement model [80]. Discriminant validity is assessed by the confidence interval test, which involves calculating the 95% confidence interval around the correlation between the factors. If the 95% confidence interval is not higher than the square root of the AVE, discriminant validity is demonstrated. The results of each pair of dimensions in this study are shown in Table 4. Discriminant validity for these constructs is supported. The assessment of the measurement model supported the reliability and validity.

### 4.3. Experience of LDCW

The CFA results are reported in Table 5. The experience of long-distance collective walkers includes three aspects of the well-being experiences: physical and emotional experience, social experience, and environmental experience. Tests of Hypothesis 1 showed that compared to individual or small-group walking activities, long-distance outdoor walkers had more intense experiences [5,14,81]. In particular, the social interaction experience was the strongest (mean = 4.502, SD = 0.594), and the standard deviation was minimal. Following was the individual experience (mean = 4.237; SD = 0.637). The environmental experience was the lowest, but still relatively high (mean = 3.990; SD = 0.689). A paired sample T-test was used to assess statistical differences between the three scales [82]. The results suggest that the mean value of the social experience is significantly higher than the mean value of the environmental experience (*t* = 7.162, *p* < 0.01), and the mean value of the social experience is significantly higher than the mean value of the individual experience (*t* = 6.667, *p* < 0.01). There is no significant difference between the mean value of the environmental experience and the mean value of the individual experience.

Compared to general walking activities, the empirical data from the LDCW participants show a highly positive experience in social relationships. Walking is useful in helping develop social interaction, producing additional social capital through pleasant conversation with people, walking with dogs [20], meeting fellow walkers [14], and interacting with others [18]. In comparison, in competitive events such as marathons, social interaction generally only manifests between teammates [83]. LDCW participants gain support from group members, volunteers, and other participants, and the social interactions among them are more profound. In the “Shenzhen 100 km Walking” event, the participants are organized in small teams during the whole process, and the participants feel the support and encouragement from their teammates. Some participants do not participate in a team, but interact with walkers through greeting and encouraging them, sharing supplies, or supporting the volunteers and, thus, the walking event provides a social space. Participants escape from their daily environments and gain a rare social experience with friends, colleagues, and strangers just by walking. During the interviews, participants stated things like: “In the city, we usually work like a stranger, but this activity involves everyone, whether the walkers, volunteers or people around, we all seemed more cordial” and “The opportunity was provided by our company, there is little chance for colleagues to walk together like this and talk about all kinds of topics while walking.”

The second experience dimension is the individual experience. Previous studies have shown that walking generally improves physical fitness and mental health [20,84,85]. People who participate in LDCW events are more likely to challenge their physical and mental limits and achieve a sense of accomplishment, resulting in positive emotions. Furthermore, daily walking and professional events emphasize the physical experience [22], while long-distance walking emphasizes the spiritual experience [5,14]. Our study has reached similar conclusions.

Walking also provides an opportunity for walkers to engage with the environment. Environment “walkability” is a key concept influencing the feelings of well-being that arise from walking [20]. For individual long-distance walkers, the walking environment provides a bubble for a “journey of self-discovery” [15]. In LDCW events, people are not just concerned about environmental walkability, and the connections with multiple environments provide a meaningful environmental experience for participants, including the intrinsic value of both city life [1,86] and rural nature [2]. People move through the walking environment to deepen their understanding of the living environment, think about the meaning of walking in the city they live in, and thereby achieve a sense of well-being.

### 4.4. The Moderating Effect of Personality Traits and Familiarity

The third objective of the study was to test the moderating effect of personality traits and familiarity. The participants were categorized into two types: highly adventurous and less adventurous. Participants agreed that both enjoying outdoor activities and self-challenges are defined as highly adventurous. In terms of familiarity, the participants were clustered into two types: high familiarity and low familiarity. The high familiarity participants were defined by whether they had enrolled for the LDCW in advance.

To examine whether personality traits and familiarity influenced the LDCW experience, multivariable analysis of variance (MANOVA) was used [87]. Table 6 and Table 7 show the summary of the multivariate and univariate results. MANOVA results suggest that the main effects of all three moderators on the three experiential dimensions are significant (*F* = 22.435, *p* < 0.01; *F* = 3.736, *p* < 0.01; *F* = 2.250, *p* < 0.05). The interaction effect of familiarity and gender on experience is significant (*F* = 2.538, *p* < 0.01). This partly supports Hypotheses 2–4.

**Figure 3:**
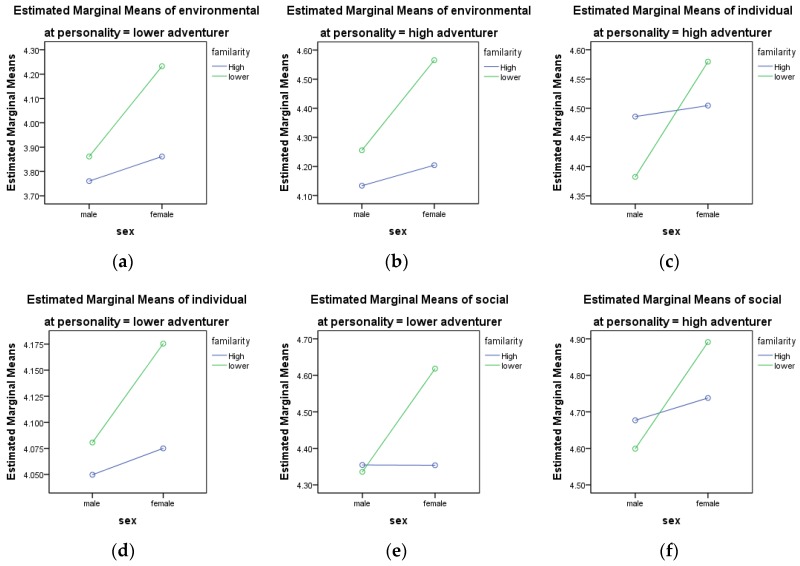
Interactive influence of personal traits and familiarity to walking experience.

**Environmental experience**. A univariate analysis indicated that all three factors had significant effects on the environmental experience. The F values are 36.785, 8.201, and 4.631, respectively, for the factors of adventurous, familiarity, and gender, and the *p*-values are all lower than 0.01. There is a significant mean difference between high adventurous and low adventurous (mean difference = 0.366, *t*(924) = 8.273, *p* < 0.01). Hypothesis 3 was then tested. Female participants’ environmental experience is significantly higher than male participants’ environmental experience (mean difference = 0.138, *t*(880) = −2.682, *p* < 0.01). Hypothesis 7 testing showed that participants who were less familiar with LDCW had a significantly higher environmental experience than participants who were more familiar with LDCW (mean difference = −0.178, *t*(881) = −3.241, *p* < 0.05). When hypothesis 11 was tested, the interaction effect of familiarity and sex on the environmental experience was significant (*F* = 1.958, *p* < 0.05). Female participants with lower familiarity (M = 4.565) had a significantly higher environmental experience than other participants. This interaction effect is further illustrated in Figure 3.

**Individual experience**. A univariate analysis indicated that the factor adventurous had significant effects on the individual experience (*F* = 52.376, *p* < 0.01). There is a significant mean difference between high adventurous and low adventurous. Participants with higher adventurousness (M = 0.201) had a significantly higher individual experience than the participants with lower adventurousness (M = 3.843). Hypothesis 4 was also supported. The mean difference was −0.426 (*t*(924) = −10.628, *p* < 0.01). There was no interaction effect.

**Social interaction experience**. A univariate analysis indicated that all three factors had significant effects on the social interaction experience. The F values are 38.569 (*p* < 0.01), 2.432 (*p* < 0.1), and 3.99 (*p* < 0.01). There is a significant mean difference between high adventurous and low adventurous (mean difference = 0.324, *t*(924) = 8.499, *p* < 0.01). Hypothesis 5 was then confirmed. Female participants’ social interaction experience was significantly higher than male participants’ social interaction experience (mean difference = −0.07, *t*(880) = −1.659, *p* < 0.1). Tests of Hypothesis 9 showed that the interaction effect of familiarity and sex on the social interaction experience was significant (*F* = 6.525, *p* < 0.01). Female participants with lower familiarity (M = 4.891) had a significantly higher social interaction experience than others. This interaction effect is further illustrated in Figure 3.

The results support these hypotheses; higher adventurous participants have a higher experience in each dimension. These results show that personality traits are not just a factor in decision-making processes [52,88], but they also influence the overall experience when participating in a leisure activity.

These results support the hypothesis that walkers’ gender affects the experiences of well-being. Specifically, female walkers obtain well-being from social interaction and individual development more than male walkers. Such results may be due to the historical perception of walking and the different cognitions of LDCW. Walking has also been considered an acceptable form of physical activity for women as it was perceived to be consistent with femininity [57]. However, LDCW was seen as a competitive sport for men, which is more in line with their need for physical activity or mental health. At the same time, women walk more as a leisure (or recreational) activity to gain well-being through contact with new environments and different people.

Furthermore, participants with lower familiarity had intense environmental and social experiences. Generally, familiarity can be treated as a kind of positive and active prior accumulated experience [63]. However, according to construal level theory, familiarity may just increase customers’ perception of the core value provided by a product’s or service’s quality [62]. A study from the perspective of destination image attributes showed that pre-trip planning would turn a destination from fantasy to reality [75]. For walking event participants, the social interaction experience as the periphery experience is high when participants are less familiar with LDCW. This is the typical character of collective physical activity in China. People do not need to engage in a lot of pre-planning behavior, and they may have high well-being experiences.

## 5. Conclusions 

### 5.1. Summary of Findings

The traditional focus of walking promotion campaigns has involved beliefs about the benefits of walking on physical health [48], but walking is now more regarded as a recreational activity which may enhance well-being [20,89]. LDCW, as a booming walking activity in China, has scarcely been explored. The current study has the strength of being the first quantitative exploration of well-being experiences and related influential factors of LDCW in the context of China framed by the theory of therapeutic mobilities and construal level theory. Therapeutic mobilities theory provides a base for developing experience scales. Construal level theory is applied to examine the influential factors. Therapeutic mobilities theory posits that the walking experience helps participants in walking toward well-being. Construal level theory asserts that the walking experience is filled with dynamics. This finding provides an empirical demonstration of Gatrell’s (2013) therapeutic mobilities theory, which suggests that walking improves health and well-being through the active body, social body, and walking contexts [20]. The finding is consistent with construal level theory.

Based on the “Shenzhen 100 km Walking” event, the following conclusions are obtained:

(1) The participants gain well-being from three dimensions: social interaction experience, individual experience, and environmental experience.
(2) In LDCW, walkers with higher adventure-seeking personality traits have more well-being experiences in each dimension.
(3) Gender has a significant influence on both the environmental experience and the social interaction experience, and female walkers obtain well-being from social interaction and individual development more than male walkers while males have more experience of individual development.
(4) Familiarity has a significant influence on both the environmental experience and the social interaction experience, and participants with less familiarity have significantly more environmental experiences.
(5) Gender and familiarity also have a significant interaction influence on both the environmental experience dimension and the social interaction experience dimension. Female participants with lower familiarity have stronger environmental experience and social experience.

This study provides several theoretical contributions, including on the walking experience, well-being, and event management.

First, this paper provides a new perspective to understand a walking event based on the participants’ experience. Following systematic scale development procedures, this paper developed and validated a measurement scale to identify LDCW participants’ social interaction, individual development, and environmental understanding experiences. Although walking experience research has been a hot topic in recent years [90,91], a limitation of former research is the lack of quantitative measurements of walkers’ experience from the perspective of well-being and therapeutic benefits. This study bridges this research gap by validating that walking participants generally have rich experiences that enhance their well-being.

Second, this paper contributes to construal level theory by testing the experiences’ influential factors, such as adventure-seeking personality, familiarity, and gender. According to construal level theory, different dimensions of psychological distance affect mental construals [49]. This paper is the first attempt to test multilevel influential factors in experience research. The findings suggest that experiences are affected by multiple psychological dimensions; this provides a new perspective to explore complex mental construals.

### 5.2. Implications

Several practical implications can be derived from this study. Contemporary Chinese are keen for group leisure and fitness activities, and long-distance walking is an effective way to achieve the goal of national fitness. This is a topic that has important policy implications for public health domains. Participants are motivated to engage in more physical exercise, while obtaining happiness and health. Therefore, proactive policies should be formulated to encourage and manage these activities. Based on the findings, the following implications can be provided for event organizers: First, they should be mindful to provide a diverse walking environment, opportunities for social interaction, physical exercise, and mental challenges, so that participants can gain well-being and events can attract more people to participate. Second, they should focus more on participants’ social interaction rather than competition. Also, organizers can promote participants’ environmental experiences and enhance their urban identities by arranging different routes, designing different themes, and creating different activities. Third, event organizers should cater to the needs of different people with different personality traits. For example, female participants should be given more opportunities to communicate in the group, while male participants should be challenged more in the routes. Participants with high familiarity should maintain consistency in the innate character of the event while innovating in other dimensions.

### 5.3. Limitations and Future Research

This research also has some limitations. First, the study collected data only from the “Shenzhen 100 km Walking” event, and whether the conclusions of this study can be extended to other walking events such as the official organization of competitive long-distance walking events needs further examination. Second, the survey sample was mostly well educated, young individuals, so the results might not be generalizable to other populations. In the meantime, having more than half the questionnaires collected at the end of the walk might have led to a more expert sample of walkers, who actually managed to finish the walk. Third, diverse psychological dimensions might affect participants’ experience. The influential factors, such as familiarity, also need more detailed exploration.

The dynamics and experiences gained during the walking process are worth additional investigation [14]. Further quantitative and/or qualitative (including mixed methods) research can explore more details of the relationships between walking and well-being. Future research is also warranted on the different types of LDCW activities that can support health and well-being, while looking at different cultural contexts and different socio-economic groups.

## Figures and Tables

**Table 1:** Walking experience scales.

Construct and Items	Sources
**Physical and Emotional aspects**	
bring health and fitness	[22]
to achieve a reflexive awareness of the self	[2]
overcoming physical challenge	[15]
get psychological benefits	[15]
overcoming psychological challenge	[15]
get a new vision of life	interview
embrace trade-offs and compromise	interview
gain achievement	[76]
stress release	[14,18]
**Environmental aspects**	
get to know new places	[76]
feeling a closeness with nature	[14]
enjoy beautiful scenery	[14,76]
experience city culture	interview
to understand city condition	interview
perceived meaning of environmental protection	interview
**Social aspects**	
get help from volunteer	[18]
support within the team member	[18]
encouragement among participants	[14,76]
interaction with others/huts	[18]

**Table 2:** Exploratory factor analysis for walking event participants.

Factor	Skewness	Kurtosis	MV(SD)	Factor Loading	Cronbach’s α
**Environmental experience**				25.196% ^a^	0.878
3.4 experience city culture of Shenzhen	−1.136	1.698	4.17 (0.884)	0.861	
3.6 perceived meaning of environmental protection	−1.292	1.542	4.18 (0.829)	0.832	
3.2 enjoy beautiful scenery	−0.676	0.031	3.98 (0.98)	0.754	
3.3 feeling a closeness with nature	−0.882	0.901	4.09 (1.05)	0.718	
3.5 to understand city condition of Shenzhen	−0.363	−0.6	3.9 (0.954)	0.71	
3.1 get to know new places	−1.589	2.369	4.26 (1.016)	0.512	
**Activity experience**				20.672% ^a^	0.843
2.3 overcoming psychological challenge	−1.351	1.947	4.38 (0.823)	0.844	
1.3 overcoming physical Challenge	−1.704	2.296	4.5 (0.82)	0.803	
2.1 get psychological benefits	−1.053	0.53	4.45 (0.7)	0.699	
1.2 to achieve a reflexive awareness of the self	−0.668	−0.734	4.2 (0.883)	0.602	
2.2 get a new vision of life	−0.952	−0.071	4.57 (0.572)	0.594	
2.4 gain achievement	−0.597	−0.884	4.17 (0.895)	0.564	
**Social interaction experience**				20.32% ^a^	0.869
4.3 encouragement among participants	−1.691	2.345	4.61 (0.663)	0.921	
4.2 support within the team member	−1.801	2.529	4.63 (0.674)	0.873	
4.4 interaction with others/huts	−1.287	0.324	4.56 (0.684)	0.789	
4.1 get help from volunteer	−1.692	1.978	4.57 (0.753)	0.706	
**Cumulative validity**				66.188% ^a^	

^a^ Denotes for variance contribution rate.

**Table 3:** The demographic characteristics of the respondents.

Items	Sample size	Proportion (%)
**Gender**		
Male	666	72.3
Female	255	27.7
**Age**		
26 and under	324	34.0
27–35	393	42.5
36–45	141	15.2
46–59	61	6.6
60 and above	6	0.6
**Education**		
Primary school and below	8	0.9
Junior middle school	26	2.8
High school	128	12.8
Training school	263	28.4
Bachelor degree and above	500	54.0
**Occupation**		
Government/institution	68	7.4
State-owned enterprise	130	14.1
Private enterprise	497	53.8
Self-employed person	46	5.0
Student	55	6.0
Retired	5	0.5
Agriculture	5	0.5
Other	118	12.8

**Table 4:** Discriminant validity test of constructs (95% confidence interval of correlates).

	Environmental		Activity	
	Lower Bound	Upper Bound	Lower Bound	Upper Bound
Activity	0.452	0.559		
Social	0.402	0.522	0.333	0.467

**Table 5:** Confirmatory factor analysis for walking event participants.

Factor	MV (SD)	Factor Loading	Std. Factor Loading	CR	Average Extracted Variance
**Environmental experience**	3.99 (0.69)		0.870 ^b^	0.871	0.530
3.4 experience city culture of Shenzhen	3.98 (0.925)	0.781	0.798		
3.3 feeling a closeness with nature	3.96 (0.886)	0.772	0.755		
3.5 to understand city condition of Shenzhen	3.92 (0.903)	0.754	0.741		
3.2 enjoy beautiful scenery	4.05 (0.856)	0.704	0.670		
3.1 get to know new places	3.84 (0.891)	0.683	0.681		
3.6 perceived meaning of environmental protection	4.19 (0.862)	0.672	0.717		
**Individual experience**	4.23 (0.68)		0.843 ^b^	0.847	0.480
2.3 overcoming psychological challenge	4.26 (0.879)	0.821	0.761		
1.3 overcoming physical Challenge	4.38 (0.846)	0.775	0.674		
2.1 get psychological benefits	4.35 (0.787)	0.728	0.756		
2.4 gain achievement	4.09 (0.951)	0.661	0.628		
1.2 to achieve a reflexive awareness of the self	4.27 (0.800)	0.598	0.621		
2.2 get a new vision of life	4.08 (0.855)	0.593	0.706		
**Social interaction experience**	4.50 (0.60)		0.855 ^b^	0.858	0.603
4.3 encouragement among participants	4.54 (0.708)	0.832	0.842		
4.2 support within the team member	4.46 (0.740)	0.808	0.815		
4.4 interaction with others/huts	4.51 (0.690)	0.784	0.764		
4.1 get help from volunteer	4.50 (0.716)	0.712	0.676		

CR: Composite Reliability; Factor loadings of items on factors to which they belong; ^b^ Cronbach alpha.

**Table 6:** MANOVA results for experience differences under the influence of personal traits and familiarity.

Dependent Variable	Source	Type III Sum of Squares	d*f*	Mean Square	*F*	Sig.
**Multivariate Statistics**	adventurous		3		22.435	0.000
	Familiarity		6		3.736	0.001
	Gender		9		2.250	0.017
	adventurous * Familiarity		3		0.385	0.764
	adventurous * Gender		3		0.234	0.872
	Familiarity * Gender		3		2.538	0.055
	adventurous * Familiarity * Gender		3		0.220	0.882
**Univariate Statistics**						
**Environmental experience**	Model	14,023.821 ^a^	11	1274.893	2926.739	0.000
	adventurous	16.023	1	16.023	36.785	0.000
	Familiarity	7.145	2	3.572	8.201	0.000
	Gender	6.052	3	2.017	4.631	0.003
	adventurous * Familiarity	0.000	1	0.000	0.001	0.978
	adventurous * Gender	0.059	1	0.059	0.137	0.712
	Familiarity * Gender	1.958	1	1.958	4.494	0.034
	adventurous * Familiarity * Gender	0.008	1	0.008	0.019	0.890
**Individual experience**	Model	15,879.146 ^b^	11	1443.559	3935.115	0.000
	adventurous	19.214	1	19.214	52.376	0.000
	Familiarity	0.618	2	0.309	0.843	0.431
	Gender	0.965	3	0.322	0.877	0.453
	adventurous * Familiarity	0.158	1	0.158	0.430	0.512
	adventurous * Gender	0.049	1	0.049	0.134	0.714
	Familiarity * Gender	0.534	1	0.534	1.455	0.228
	adventurous * Familiarity * Gender	0.073	1	0.073	0.199	0.656
**Social interaction experience**	Model	17,894.661 ^c^	11	1626.787	5058.896	0.000
	adventurous	12.403	1	12.403	38.569	0.000
	Familiarity	1.558	2	0.779	2.423	0.089
	Gender	3.858	3	1.286	3.999	0.008
	adventurous* Familiarity	0.201	1	0.201	0.625	0.430
	adventurous * Gender	0.030	1	0.030	0.094	0.759
	Familiarity * Gender	2.098	1	2.098	6.525	0.010
	adventurous * Familiarity * Gender	0.051	1	0.051	0.158	0.691

^a^. R Squared = 0.974 (Adjusted R Squared = 0.973); ^b^. R Squared = 0.980 (Adjusted R Squared = 0.980); ^c^. R Squared = 0.985 (Adjusted R Squared = 0.984)

**Table 7:** MANOVA summary statistics.

	Higher Familiarity				Lower Familiarity			
	Male		Female		Male		Female	
Environmental experience	3.925	(0.702)	3.998	(0.685)	4.024	(0.654)	4.358	(0.566)
higher adventurous	4.134	(0.728)	4.205	(0.671)	4.256	(0.630)	4.565	(0.596)
lower adventurous	3.760	(0.635)	3.861	(0.662)	3.861	(0.625)	4.232	(0.515)
Individual experience	4.242	(0.648)	4.246	(0.640)	4.205	(0.612)	4.328	(0.606)
higher adventurous	4.486	(0.588)	4.505	(0.555)	4.382	(0.514)	4.580	(0.495)
lower adventurous	4.050	(0.628)	4.075	(0.637)	4.081	(0.647)	4.175	(0.621)
Social interaction experience	4.497	(0.609)	4.507	(0.595)	4.444	(0.581)	4.721	(0.377)
higher adventurous	4.677	(0.552)	4.738	(0.428)	4.599	(0.466)	4.891	(0.224)
lower adventurous	4.355	(0.615)	4.354	(0.641)	4.336	(0.630)	4.618	(0.414)

Standard deviations are shown within parentheses.
